# Digital Health Interventions Targeting Psychological Health in Parents of Children With Autism Spectrum Disorder: Protocol for a Scoping Review

**DOI:** 10.2196/68677

**Published:** 2025-06-04

**Authors:** Binbin Ji, Intan Maharani Sulistyawati Batubara, Janene Batten, Xinyi Peng, Sanmei Chen, Zhao Ni

**Affiliations:** 1 School of Nursing Hunan University of Chinese Medicine Changsha China; 2 School of Nursing Yale University New Haven, CT United States; 3 Harvey Cushing/John Hay Whitney Medical Library Yale University New Haven, CT United States; 4 Division of Nursing Graduate School of Biomedical and Health Sciences Hiroshima University Hiroshima Japan; 5 Yale Institute for Global Health Yale University New Haven, CT United States

**Keywords:** autism spectrum disorder, digital health intervention, parents, psychological health, scoping review

## Abstract

**Background:**

Autism spectrum disorder (ASD) is a complex, incurable condition requiring lifelong care, often placing significant psychological strain on parents and emerging as a public health concern. While various interventions exist to enhance the psychological health of parents, the role of digital health interventions (DHIs) in this context remains underexplored.

**Objective:**

This scoping review aims to systematically assess the availability of DHIs targeting the psychological health of parents of children with ASD and evaluate the effectiveness of these interventions in improving parental psychological health.

**Methods:**

The review will include English-language studies published from inception to June 25, 2024, focusing on DHIs aimed at improving the psychological health of parents of children with ASD. Eligible studies will involve parents of children with ASD less than the age of 18 years, across various settings, and assess psychological health outcomes. A comprehensive search will be conducted across six databases: (1) CINAHL, (2) Ovid EMBASE, (3) Ovid Global Health, (4) Ovid MEDLINE, (5) Ovid PsycINFO, and (6) Web of Science. Studies will be screened and selected based on predefined eligibility criteria. Data extraction will include publication details, study design, participants’ characteristics, intervention specifics, comparisons, psychological outcomes, and key findings. Results will be synthesized using descriptive statistics, charts, and narrative analysis.

**Results:**

The initial keyword-based search, completed in June 2024, identified 5825 records, which were subsequently screened and analyzed. Screening and evidence synthesis were finalized in winter 2024, and the completed scoping review was submitted in December 2024.

**Conclusions:**

This study will provide a comprehensive overview of commonly used DHIs for supporting the psychological health of parents of children with ASD and their effectiveness. The findings will help identify research gaps, inform future studies and funding priorities, and contribute to the development of practice guidelines to enhance parental psychological health.

**International Registered Report Identifier (IRRID):**

DERR1-10.2196/68677

## Introduction

### Background

Autism spectrum disorder (ASD) is a lifelong neurodevelopmental condition characterized by early-onset difficulties in social communication, repetitive and stereotyped behaviors, and narrow interests [1]. According to the Autism and Developmental Disabilities Monitoring (ADDM) Network program, approximately 1 in 36 children are identified with ASD [2]. Despite extensive research, ASD remains a complex neurodevelopmental disorder with an unclear etiology and no definitive cure [3]. While children with ASD generally have a normal life expectancy, they often require lifelong care, particularly from their parents, who typically serve as primary caregivers [4,5]. The demands of caregiving for a child with ASD are significant and can negatively affect the psychological health of parents.

A growing body of evidence indicates that parents of children with ASD are at a higher risk for mental health issues compared to those raising children with other developmental disabilities [6-8]. Studies consistently show that caregiving for a child with ASD is associated with elevated stress levels, negative emotional states, and symptoms of depression and anxiety [8-10]. These psychological difficulties can deplete both parents' and children’s coping energies and impair their capacity for problem-solving [11]. The impact of ASD caregiving on parental psychological health has emerged as a critical public health concern, emphasizing the need for targeted interventions.

While considerable emphasis has been placed on providing high-quality treatment and care to children with ASD, less attention has been paid to the psychological health of their parents. Previous research has explored interventions aimed at improving outcomes for children with ASD, as well as for their parents [12,13]. However, many of these studies primarily focus on training parents to promote their child’s social skills, communication, and learning [14,15], with relatively few studies directly addressing parental health. Improving the psychological health of parents is critical, not only for their well-being but also for enhancing outcomes for their children. Therefore, there is a need for innovative interventions to support and train parents, especially considering the increasing role of digital technologies in health care delivery.

The rapid advancement of digital health technologies offers significant potential for addressing these gaps. During the COVID-19 pandemic, digital health interventions (DHIs) were widely adopted as a supplement to conventional health services [16,17]. Although the definitions of digital health vary, there is a consensus that DHIs, including computer-based therapies, mobile apps, and wearable devices, can enhance the accessibility, efficiency, and personalization of mental health care [18]. Evidence suggests that digital technologies can empower individuals and families affected by mental health issues, offering convenience, privacy, and opportunities for independent learning [19,20]. These features make DHIs particularly attractive for supporting parents of children with ASD [21,22].

To develop effective DHIs for parents of children with ASD, it is essential to understand which interventions have been developed, tested, and implemented. Several studies have examined the potential benefits of DHIs in reducing parenting stress and enhancing self-efficacy [23,24]. However, most studies have primarily focused on interventions targeting children with ASD, with relatively little attention given to the health of parents [25,26]. A recent review of interventions for parents of older children and adults with ASD highlighted the potential benefits of parent-focused interventions in improving psychological health [27], but it did not address the role of DHI.

This scoping review aims to fill this gap by examining the availability of DHIs designed to support the psychological health of parents and evaluating their effectiveness. It is part of a broader study focused on DHIs aimed at enhancing psychological health among parents of children with ASD. By identifying gaps in the current literature, this review will provide a foundation for the future development of DHIs specifically tailored to the needs of parents. No prior scoping or systematic reviews on this specific topic were identified, making this review an important contribution to the field.

### Review Questions

This review will focus on answering 4 key research questions. First, which DHIs have been developed and implemented to support parents of children with ASD? Second, how are these interventions specifically designed to improve the psychological health of these parents? Third, what are the primary psychological health outcomes associated with these interventions? Fourth, what gaps, limitations, and opportunities exist for the future development of DHIs aimed at supporting the psychological health of parents of children with ASD?

## Methods

### Overview

The scoping review will be conducted in accordance with the methodology established by the Joanna Briggs Institute (JBI). Articles will be screened and selected using the PRISMA-P (Preferred Reporting Items for Systematic Reviews and Meta-Analyses Protocols), and the results of the review will be presented according to the PRISMA-ScR (Preferred Reporting Items for Systematic Reviews and Meta-Analyses extension for Scoping Reviews) [28] (Multimedia Appendix 1). To report the functional classification of the final DHIs selected for review, we will use the Evidence Standards Framework for Digital Health Technologies developed by the UK National Institute for Health and Care Excellence (NICE) [29]. The format of this protocol adheres to the author guidelines of the target publication and is informed by recommendations regarding items to be reported in the scoping review [30,31].

### Eligibility Criteria

Researchers use the Population, Intervention, Comparator, and Outcomes (PICO) framework to identify the key concepts of a scoping review, develop appropriate search terms to describe the problem, and determine inclusion and exclusion criteria.

#### Population

In our review, we define parents as biological parents, birth parents, or foster parents of children with ASD younger than 18 years. The World Health Organization (WHO) defines children as people younger than 18 years [32]. This age range has been restricted because we are focused on reviewing DHIs that support parents during the early years of their children’s development. Studies reporting on DHIs for parents of children older than 18 years will be excluded.

#### Intervention

This review will include interventions that use communication media for DHI. DHIs, including computer-based therapies, mobile apps, and wearable devices, can enhance the accessibility, efficiency, and personalization of mental health care [18]. This review will include computer-assisted therapy, smartphone apps, and wearable technologies that support parents’ psychological health. Studies that do not focus on digital technologies will be excluded.

#### Comparator

Comparators or comparisons refer to groups that were compared to the intervention group [33]. The comparison can be to another treatment or intervention or no treatment at all. In this scoping review, all articles will be considered for inclusion, regardless of whether they have a control group or the type of control used.

#### Outcomes

Outcome refers to the measure of interest or the study’s target endpoints that are objectively defined, essentially representing the variable being assessed to determine the effect of an intervention or exposure on a population [34]. In this scoping review, the articles must include the outcomes on parental psychological health (stress, anxiety, depression, distress, fatigue, quality of life, positive thinking, happiness, family empowerment, hope, resilience, etc), which will be assessed using validated psychological scales and measures of subjective well-being.

#### Types of Studies

This scoping review will consider qualitative, randomized controlled trials, cohort studies, case-control studies, cross-sectional surveys, pre- and postintervention studies, mixed methods studies, and observational studies. Studies that are not peer-reviewed, gray literature, nonempirical studies, review articles, conference papers, preprints, or limited to abstracts will be excluded. This review will consider studies conducted or implemented to support parents’ psychological health in community settings, school settings, special schools, clinics, hospitals, at home, and child development centers. There will be no exclusion based on region, gender, and sociocultural factors.

### Search Strategy

The search strategy was developed in collaboration with an expert medical librarian (JB) and 2 researchers (BJ and ZN). We will conduct our search across 6 bibliographic databases: (1) CINAHL, (2) Ovid EMBASE, (3) Ovid Global Health, (4) Ovid MEDLINE, (5) Ovid PsycINFO, and (6) Web of Science. These databases were selected based on their relevance to the research topic and their comprehensive coverage of the literature in this field. Each database will be searched using both controlled vocabulary and synonymous free-text terms to encompass the concepts of autism and digital health. The search strategy will be tailored to accommodate the syntax specific to each database. Inclusion will be limited to English-language studies, with no restrictions on publication date, from inception to June 25, 2024. Search results will be imported into EndNote (version 20, Clarivate), and duplicate entries will be removed. The final set of included articles will then be uploaded into Covidence systematic review software (Veritas Health Innovation) for screening. Detailed search histories can be found in Multimedia Appendix 2.

### Study Selection

Following the search, all identified records will be uploaded into a data screening sheet created in Covidence (Veritas Health Information), where duplicates will be removed. Subsequently, 3 researchers (BJ, IMSB, and XP) will screen the titles and abstracts of all retrieved articles using Covidence, assessing the full texts for eligibility based on predefined inclusion and exclusion criteria. Discrepancies will be resolved through discussion with the senior author (ZN). The reasons for exclusion will be documented for articles eliminated following full-text screening. See Multimedia Appendix 3 for the PRISMA (Preferred Reporting Items for Systematic Reviews and Meta-Analyses) flow diagram of the literature search and selection process.

### Data Extraction

A summary of the findings from the included studies will be presented in a table using a data extraction form (Multimedia Appendix 4). This form will capture essential information from the eligible studies, including the authors, publication year, study locations (country), study design, participants, interventions (including name, digital technologies, duration, etc), comparisons, psychological outcomes for parents, and key findings.

### Data Analysis and Presentation

We will perform a descriptive analysis to aggregate and synthesize data segments that demonstrate similarities. Deductive coding will be used to categorize and analyze data in line with the identified outcomes from the included studies. Interrater reliability will be assessed to ensure the consistency and accuracy of the coding process. The data extracted from the included studies will be systematically tabulated and summarized narratively. Through narrative synthesis, the analysis organizes findings around recurring themes or clusters, such as types of digital interventions (eg, apps vs wearable devices) and common psychological outcomes (eg, stress reduction and resilience building). The descriptive synthesis also highlights areas with limited research or emerging needs, revealing gaps in interventions targeting specific psychological outcomes or identifying underrepresented study populations and settings.

### Ethical Considerations

There will be no involvement of humans, animals, patients, or the public in this review. This review will synthesize data from peer-reviewed studies that have been published. Thus, ethical approval is not required for this scoping review.

## Results

The electronic database search was completed in June 2024, marking the beginning of the research team’s familiarization with DHI and the psychological health of parents of children with ASD. During this stage, the team consulted with a medical librarian (JB) at Yale University regarding search strategies and preliminary results. A total of 5825 records were retrieved, and 2296 were identified as duplicates. Full-text screening was conducted in September and October 2024. Data extraction and thematic synthesis were carried out in winter 2024. At the time of submission, the study was in the data analysis phase. The scoping review summarizes findings including population characteristics, study design, participant demographics (number and age), DHI details (name, type, platform/tool, duration), comparisons, psychological outcomes (variables and measurement tools), and key findings (significant changes, group differences, and follow-up effects). The review was submitted for publication in December 2024.

## Discussion

This scoping review will explore the existing evidence on the use of DHIs to support the psychological health of parents of children with ASD. Through this review, we aim to identify gaps, limitations, and opportunities in existing knowledge to inform the development of future DHIs. The scoping review protocol presented in this paper is novel. To the best of our knowledge, no previous reviews have specifically focused on DHIs for the psychological health of parents of children with ASD [19,35]. Reviews that examine DHIs in the context of the ASD population have generally concentrated on children with ASD rather than their parents [36,37]. This scoping review will not only identify gaps in the literature but also provide valuable insights to guide future research and the development of practice guidelines aimed at supporting the psychological health of parents of children with ASD.

Psychological health is a complex interplay of mental, emotional, social, and spiritual dimensions of well-being [38]. Like physical health, it is a crucial component of overall wellness. Parenting a child with ASD presents unique challenges that can adversely affect the psychological health of parent carers. Parents of children with ASD are at an elevated risk for negative psychological outcomes, including depression, anxiety, stress, and emotional distress [8,10,39,40]. When a parent’s psychological health is compromised, it can hinder their ability to make decisions, regulate emotions, manage behaviors, engage in social interactions, and cope with stress [41]. Moreover, children of parents who experience heightened psychological challenges often experience various negative psychological and developmental outcomes [42,43]. Therefore, supporting parental psychological health is not only crucial for their well-being but also for fostering healthier developmental outcomes in their children.

Parent training and support have long been regarded as important components of early intervention programs for children with ASD. Empirical evidence indicates that parent-focused training and support programs can positively impact children’s behavior and functional communication skills, leading to improved parent-child interactions and enhanced psychological health for parents [44,45]. However, traditional in-person interventions for parents of children with ASD face significant limitations due to constraints such as time, location, cost, and accessibility. With the rapid advancements in digital health technologies and the onset of the COVID-19 pandemic, DHIs have become increasingly used in this domain. Nevertheless, most applications of DHIs have occurred in developed countries, and findings regarding their impact on the psychological health of parents of children with ASD have been mixed [19,46-48].

The results of this study will provide a complete picture of the types of DHIs used to improve parental psychological health and their effectiveness. DHIs have been shown to positively influence psychological health [49,50]. Therefore, the integration of digital technologies into interventions may help reduce psychological health problems among parents. The findings of this scoping review will serve as a basis for further research or intervention development.

This scoping review will provide an overview of the existing literature on the use of DHIs to improve the psychological health of parents of children with ASD. One limitation of this review is the exclusion of non-English studies, which may limit the generalizability of findings. In addition, unpublished studies, such as conference abstracts, dissertations, and gray literature, were not included, potentially omitting relevant insights. As a scoping review, this study aims at mapping the available literature rather than assessing the effectiveness of interventions. Future research will build on this work with a quantitative meta-analysis to assess the impact of DHIs on the psychological health of parents of children with ASD. Our dissemination plan includes presentations at conferences and for relevant stakeholders, and publication in a peer-reviewed journal.

## Appendix

Multimedia Appendix 1 PRSIMA-ScR (Preferred Reporting Items for Systematic Reviews and Meta-Analyses extension for Scoping Reviews) checklist.

Multimedia Appendix 2 Search strategy.

Multimedia Appendix 3 PRISMA (Preferred Reporting Items for Systematic Reviews and Meta-Analyses) flow diagram.

Multimedia Appendix 4 Data extraction instrument.

## Abbreviations

ADDM: Autism and Developmental Disabilities Monitoring Network
ASD: autism spectrum disorder
DHI: digital health interventions
JBI: Joanna Briggs Institute
NICE: UK National Institute for Health and Care Excellence
PICO: Population, Intervention, Comparator, Outcomes
PRISMA: Preferred Reporting Items for Systematic Reviews and Meta-Analyses
PRISMA-P: Preferred Reporting Items for Systematic Reviews and Meta-Analysis Protocols
PRISMA-ScR: Preferred Reporting Items for Systematic Reviews and Meta-Analyses extension for Scoping Reviews
WHO: World Health Organization
