# Supplementary material for: Digital Health Interventions Targeting Psychological Health in Parents of Children With Autism Spectrum Disorder: Protocol for a Scoping Review
Source: JMIR Res Protoc. 2025 Jun 4;14:e68677. doi: 10.2196/68677 (PMC12177426; doi:10.2196/68677)
Supplement: Multimedia Appendix 2 [file resprot_v14i1e68677_app2.docx]

**Appendix 2: Search Strategy**

Ovid MEDLINE (R) ALL

| 1 | exp Telemedicine/ |
| --- | --- |
| 2 | exp Digital Health/ or exp Internet/ or exp Mobile Applications/ or exp Telemedicine/ or exp Cell Phone/ or exp Smartphone/ |
| 3 | (Smartphone* or App or apps or WeChat or Text* or Virtual* or telehealth or digital or cellphone* or cell phone*).ti, ab, kw. |
| 4 | 1 or 2 or 3 |
| 5 | (autism or autistic).mp. or exp Autistic Disorder |
| 6 | 4 and 5 |
| 7 | exp Parents/ |
| 8 | exp Fathers |
| 9 | exp Mothers/ |
| 10 | exp Caregivers |
| 11 | exp Family/ |
| 12 | (parent* or father* or mother* or caregiver* or carer* or family or families).ti, ab, kw. |
| 13 | 7 or 8 or 9 or 10 or 11 or 12 |
| 14 | 6 and 13 |
| 15 | review.m_title. |
| 17 | 14 not 15 |

OVID Embase

| 1 | exp telemedicine/ |
| --- | --- |
| 2 | exp digital technology |
| 3 | exp Internet/ |
| 4 | exp mobile application |
| 5 | exp telemedicine/ |
| 6 | exp mobile phone |
| 7 | exp smartphone |
| 8 | (Smartphone* or App or apps or WeChat or Text* or Virtual* or telehealth or digital or cellphone* or cell phone*).ti, ab, kw. |
| 9 | 1 or 2 or 3 or 4 or 5 or 6 or 7 or 8 |
| 10 | exp autism/ or autism.mp. or autistic.mp. [mp=title, abstract, heading word, drug trade name, original title, device manufacturer, drug manufacturer, device trade name, keyword heading word, floating subheading word, candidate term word] |
| 11 | exp parent |
| 12 | exp father |
| 13 | exp mother |
| 14 | exp caregiver |
| 15 | exp family |
| 16 | (parent* or father* or mother* or caregiver* or carer* or family or families).ti, ab, kw. |
| 17 | 11 or 12 or 13 or 14 or 15 or 16 |
| 18 | 9 and 10 and 17 |
| 19 | review.m_title. |
| 20 | 18 not 19 |
|  |  |

APA PsycInfo

|  |  |
| --- | --- |
| 1 | exp Telemedicine/ |
| 2 | exp Digital Technology/ |
| 3 | exp Internet/ |
| 4 | exp Mobile Applications/ |
| 5 | exp Telemedicine/ |
| 6 | exp Mobile Phones/ |
| 7 | exp Smartphones/ |
| 8 | (Smartphone* or App or apps or WeChat or Text* or Virtual* or telehealth or digital or cellphone* or cell phone*).ti, ab. |
| 9 | 1 or 2 or 3 or 4 or 5 or 6 or 7 or 8 |
| 10 | (autism or autistic).mp. or exp Autism Spectrum Disorders |
| 11 | exp Parents/ |
| 12 | exp Fathers |
| 13 | exp Mothers/ |
| 14 | exp Caregivers |
| 15 | exp Family/ |
| 16 | (parent* or father* or mother* or caregiver* or carer* or family or families).ti, ab. |
| 17 | 11 or 12 or 13 or 14 or 15 or 16 |
| 18 | 9 and 10 and 17 |
| 19 | review.m_title. |
| 20 | 18 not 19 |
|  |  |

OVID Global Health

|  |  |
| --- | --- |
| 1 | telemedicine |
| 2 | digital technology |
| 3 | Internet |
| 4 | mobile applications |
| 5 | telemedicine |
| 6 | mobile telephones |
| 7 | (Smartphone* or App or apps or WeChat or Text* or Virtual* or telehealth or digital or cellphone* or cell phone*).ti, ab. |
| 8 | 1 or 2 or 3 or 4 or 5 or 6 or 7 |
| 9 | (autism or autistic).mp. |
| 10 | parents |
| 11 | fathers |
| 12 | mothers |
| 13 | careproviders.sh. |
| 14 | families |
| 15 | (parent* or father* or mother* or caregiver* or carer* or family or families).ti, ab. |
| 16 | 10 or 11 or 12 or 13 or 14 or 15 |
| 17 | 8 and 9 and 16 |
|  |  |

CINAHL Complete

| S19 | S17 NOT S18 |
| --- | --- |
|  |  |
| S18 | TI "review" |
|  |  |
| S17 | S8 AND S9 AND S16 |
|  |  |
| S16 | S10 OR S11 OR S12 OR S13 OR S14 OR S15 |
|  |  |
| S15 | parent* or father* or mother* or caregiver* or carer* or family or families |
|  |  |
| S14 | (MH "Family+") |
|  |  |
| S13 | (MH "Caregivers") |
|  |  |
| S12 | (MH "Mothers+") |
|  |  |
| S11 | (MH "Fathers+") |
|  |  |
| S10 | (MH "Parents+") |
|  |  |
| S9 | (MH "Autistic Disorder") OR ("autism" OR "autistic") |
|  |  |
| S8 | S1 OR S2 OR S3 OR S4 OR S5 OR S6 OR S7 |
|  |  |
| S7 | Smartphone* or App or apps or WeChat or Text* or Virtual* or telehealth or digital or cellphone* or cell phone* |
| S6 | (MH "Smartphone") OR (MH "Text Messaging+") |
| S5 | (MH "Cellular Phone+") |
|  |  |
| S4 | (MH "Mobile Applications") |
|  |  |
| S3 | (MH "Internet+") |
|  |  |
| S2 | (MH "Digital Technology+") OR (MH "Digital Health+") |
|  |  |
| S1 | (MH "Telemedicine+") OR (MH "Telehealth+") |
|  |  |

Web of Science

| 6 | **#4 NOT #5** |
| --- | --- |
| 5 | **TI=(review)** |
| 4 | **#1 AND #2 AND #3** |
| 3 | **((((((ALL=(parent*)) OR ALL=(father*)) OR ALL=(mother*)) OR ALL=(caregiver*)) OR ALL=(carer*)) OR ALL=(family)) OR ALL=(families)** |
| 2 | **(TS=(autism)) OR TS=(autistic)** |
| 1 | **(TS=(telemedicine) OR TS=(digital health) OR TS=(internet) OR TS=(mobile application*) OR TS=(smartphone*) OR TS=(cell phone*) OR TS=(apps) OR TS=(app) OR TS=(mobile phone*) OR TS=(WeChat) OR TS=(text*) OR TS=(virtual) OR TS=(telehealth) OR TS=(digital))** |
