# Supplementary material for: Digital Health Interventions Targeting Psychological Health in Parents of Children With Autism Spectrum Disorder: Protocol for a Scoping Review
Source: JMIR Res Protoc. 2025 Jun 4;14:e68677. doi: 10.2196/68677 (PMC12177426; doi:10.2196/68677)
Supplement: Multimedia Appendix 3 [file resprot_v14i1e68677_app3.docx]

**Appendix 3** Preferred Reporting Items for Systematic Reviews and Meta-Analyses (PRISMA ) flow diagram of the literature search and selection process.

**Identification of studies via databases and registers**

Records identified from Databases searching (n=5825) :

MEDLINE (n=787)

EMBASE (n=1328)

PsycINFO (n=1122)

Global Health (n=74)

CINAHL Complete (n=775)

Web of Science (n=1739)

**Identification**

Records removed before screening (n=2296):

Duplicates identified manually (n=2119)

Duplicates identified by Covidence (n=177)

Records excluded: Title and abstract screening (n=3270)

(n = )

**Screening**

Records screened (n=3529)

Duplicate paper (n=2)

No full text (n=54)

Reports sought for retrieval

(n=259)

**Eligibility**

Full-text excluded (n=150)

Wrong study design (protocol) (n =11)

No parents of children with ASD (n=8)

Children over 18 years of age (n=2)

No digital technology reported (n=16)

No outcomes reported for parents (n=15)

Unwanted outcomes from parents (as attitudes, feasibility, etc) (n=98)

Full-text articles assessed for eligibility (n=203)

**Included**

Studies included in review

(n=53)
