# Supplementary material for: Digital Health Interventions Targeting Psychological Health in Parents of Children With Autism Spectrum Disorder: Protocol for a Scoping Review
Source: JMIR Res Protoc. 2025 Jun 4;14:e68677. doi: 10.2196/68677 (PMC12177426; doi:10.2196/68677)
Supplement: Multimedia Appendix 4 [file resprot_v14i1e68677_app4.docx]

| Appendix 4: Data extraction instrument | | | | | | | | | | | | | | |
| --- | --- | --- | --- | --- | --- | --- | --- | --- | --- | --- | --- | --- | --- | --- |
| Author (s)  (Year) | Aim or objective | Locations (Country) | Study design | Participants | Number of participants | Digital health intervention information | | | | Comparisons | Psychological outcomes for parents | Findings | | |
|  |  |  |  |  |  | Name | Type | platform and tool | Duration (weeks) |  |  | Significant change | Significant group difference | Follow-up effect (weeks) |
